# Characterization of Hair Follicle Development in Engineered Skin Substitutes

**DOI:** 10.1371/journal.pone.0065664

**Published:** 2013-06-17

**Authors:** Penkanok Sriwiriyanont, Kaari A. Lynch, Kevin L. McFarland, Dorothy M. Supp, Steven T. Boyce

**Affiliations:** 1 School of Energy, Environmental, Biological and Medical Engineering, University of Cincinnati, Cincinnati, Ohio, United States of America; 2 Department of Surgery, College of Medicine, University of Cincinnati, Cincinnati, Ohio, United States of America; 3 Research Department, Shriners Hospitals for Children, Cincinnati, Ohio, United States of America; University of Tennessee, United States of America

## Abstract

Generation of skin appendages in engineered skin substitutes has been limited by lack of trichogenic potency in cultured postnatal cells. To investigate the feasibility and the limitation of hair regeneration, engineered skin substitutes were prepared with chimeric populations of cultured human keratinocytes from neonatal foreskins and cultured murine dermal papilla cells from adult GFP transgenic mice and grafted orthotopically to full-thickness wounds on athymic mice. Non-cultured dissociated neonatal murine-only skin cells, or cultured human-only skin keratinocytes and fibroblasts without dermal papilla cells served as positive and negative controls respectively. In this study, neonatal murine-only skin substitutes formed external hairs and sebaceous glands, chimeric skin substitutes formed pigmented hairs without sebaceous glands, and human-only skin substitutes formed no follicles or glands. Although chimeric hair cannot erupt readily, removal of upper skin layer exposed keratinized hair shafts at the skin surface. Development of incomplete pilosebaceous units in chimeric hair corresponded with upregulation of hair-related genes, *LEF1* and *WNT10B*, and downregulation of a marker of sebaceous glands, *Steroyl-CoA desaturase*. Transepidermal water loss was normal in all conditions. This study demonstrated that while sebaceous glands may be involved in hair eruption, they are not required for hair development in engineered skin substitutes.

## Introduction

Engineered skin substitutes (ESS) have been recognized as an adjunctive therapy for closure of large, full-thickness wounds. As an alternative therapy to skin autografts, which may be limited by tissue availability, ESS provide functional barrier that prevents fluid loss and protects internal organs from infection. In recent years, progress has been made in developing ESS, not only to improve wound closure, but also to restore more complete anatomy and physiology of native skin. Addition of endothelial cells overexpressing vascular endothelial growth factor promotes neovascularization resulting in improved wound healing [1], [2], whereas initiation of new blood vessels can also be induced from bulge-derived, nestin-expressing hair follicles [3]. Further, involvement of hair follicles in directing nerve migration has been reported recently [3], [4]. These studies demonstrate many critical functions of skin appendages that have been overlooked for decades in the development of skin substitutes.

In contrast to other mammals, human skin appendages form completely during embryogenesis and cannot be restored in a postnatal individual after full-thickness skin loss. This limitation poses challenges in establishment of these structures in ESS. Success in hair regeneration, thus far, has depended upon the use of fresh fetal or newborn cells [5], [6]. In the present study, ESS were prepared by the use of culture expanded cells and were assessed for markers of skin, hair and sebaceous gland development, and for the factors that may regulate eruption of the hair shaft.

## Materials and Methods

### Preparation of Cultured Cells for ESS Inoculation

Murine dermal papillae (mDPC-GFP) were dissected from vibrissae of male adult C57Bl6-Tg(UBC-GFP)30 mice, and were placed into a collagen-I coated T-25 flask. Culture medium, AmnioMAX™-C100 (Life Technologies, Grand Island, NY) was changed every two days. Cells were subcultured at 2–3×10^3^cells/cm^2^ into T-225 flasks, which reached 70%–80% confluence within 5–6 days. Human neonatal foreskins were used for establishment of human keratinocytes (hK) and human fibroblasts (hF) as previously described [7]. Human skin samples used for cell cultures were obtained from neonatal foreskins, were de-identified, and were to be discarded which exempts the use of this tissue from requirements for informed consent according to 45CFR46.101(b)(4). This exempt status for collection of discard skin without informed consent was confirmed by the University of Cincinnati Institutional Review Board.

### ESS Preparation

Collagen-glycosaminoglycan (collagen-GAG) scaffolds were prepared as described elsewhere [8]. Passage 4 hF were inoculated at 5.0×10^5^cells/cm^2^. Twenty four hours later, passage 2 mDPC-GFP cells were inoculated onto the collagen-GAG containing hF at 5.0×10^5^cells/cm^2^. ESS were incubated for 9 days at air-liquid interface after hK inoculation at 1.0×10^6^cells/cm^2^. For positive controls, dissociated skin cells from newborn C57/Bl6 mice were inoculated in collagen-GAG and allowed to absorb into the dermal analog for 2 hours before grafting [9].

### Animal Studies

All studies involving animal subjects were performed under a protocol that was approved by the University of Cincinnati Institutional Animal Care and Use Committee. Methods for orthotopic grafting ESS onto athymic mice (Harlan, Indianapolis, IN) have been previously described [7], [8]. Tape stripping procedures were conducted on grafted ESS controls as well as grafted ESS with mDPC-GFP on week 5 and week 6. Sterilized pre-cut adhesive tapes were placed on the graft site to cover both ESS and some parts of murine skin before being gently peeled off. A total of 15 tapes were collected from each graft, and corneocytes were visualized under a fluorescence microscope to assure effective removal of the *stratum corneum*. Animals were euthanized 7 weeks after grafting.

### Measurement of Transepidermal Water Loss (TEWL)

Mice were anesthetized by intraperitoneal administration of Avertin, and TEWL was measured using a Tewameter integrated in an MPA5 unit (Courage and Khazaka, GmbH, Germany). Data were recorded as g/m^2^h.

### Lipid Staining

Epidermal sheets were separated after incubation at 37°C in 2 M sodium bromide for 30 minutes. Subsequently, tissues were fixed in 4% paraformaldehyde overnight at 4°C. Nile Red (0.1 µg/mL) was used to detect lipids. 4′,6-diamidino-2-phenylindole (DAPI, Life Technologies) was used to counterstain nuclei.

### Alkaline Phosphatase (ALP) Staining

Dermal sheets were fixed in 70% ethanol, washed in Tris-buffered saline (TBS), and incubated in a solution containing 100 mM Tri-HCl, 100 mM NaCl, 50 mM MgCl_2,_ pH 9.5. Thereafter, a 1∶20 dilution of 5-bromo-4-chloro-3-indoxyl phosphate/tetranitroblue tetrazolium substrate (Millipore, Billerica, MA) was applied to each specimen. After 3 hours, the reaction was stopped using a buffered solution of 20 mM EDTA.

### Immunohistochemistry

Six micron thick cryotome sections were fixed in cold acetone for 10 minutes, and were blocked for 1 hour in 3% bovine serum albumin. Primary antibodies were as follows: rabbit monoclonal anti-cytokeratin 10 (Abcam; Cambridge, MA), rabbit monoclonal anti-GFP Alexa Fluor 488 conjugated (Life Technologies), rabbit polyclonal anti-LEF1 (Cell Signaling Technology; Boston, MA), rabbit polyclonal anti-CD200 (Biorbyt, San Francisco, CA), rabbit polyclonal anti-SOX9 (Santa Cruz Biotechnology, Santa Cruz, CA), goat polyclonal anti-LHX2 (Santa Cruz Biotechnology), mouse monoclonal anti-keratin 15 (Acris Antibodies, San Diego, CA), mouse monoclonal anti-CD34 (Biorbyt), mouse monoclonal anti-Mel5 (Covance; Princeton, NJ) and mouse monoclonal anti-human nuclei (“HuNu”; Millipore) DyLight™ 549 conjugated (Pierce; Rockford, IL). Alexa Fluor 488 and/or 546 (Life Technologies) were used in indirect immunolabeling. Where applicable, nuclei were detected using DAPI (Life Technologies).

### Quantitative Real-time PCR

Total RNA was isolated using the RNeasy Mini Kit (Qiagen, Valencia, CA). Single-stranded cDNA was synthesized using SuperScript II reverse transcriptase and Oligo(dT)_12–18_ (Life Technologies). RT^2^ qPCR primer assays and SYBR Green master mix (Qiagen) were used to determine the transcript expression of *lymphoid enhancer-binding factor 1* (*LEF1*), *Wingless-type MMTV integration site family, member 10B* (*WNT10B*), *SRY (sex determining region Y)-box 9* (*SOX9*) and *PR domain zinc finger protein 1* (*PRDM1*). Taqman gene expression assays and Taqman Universal Master Mix (Applied Biosystems, Foster City, CA) were used to determine the transcript expression of *steroyl Co-A desaturase* (*SCD*), *fatty acid binding protein 3* (*FABP3*), *involucrin* (*IVL*) and *loricrin* (*LOR*). All reactions were normalized against *GAPDH* and compared to native human skin (NHS).

### Statistics

Changes in graft area over time were analyzed using repeated measures analysis of variance (ANOVA). Differences in skin TEWL were interpreted using One-Way ANOVA and Tukey’s pairwise test. Quantitative real-time PCR data were analyzed using Student’s *t*-test. Differences were considered statistically significant at the 95% confidence level (*p*<0.05).

## Results

### *In vitro* and *in vivo* Evaluation Two ESS Models

**Figure 1:**
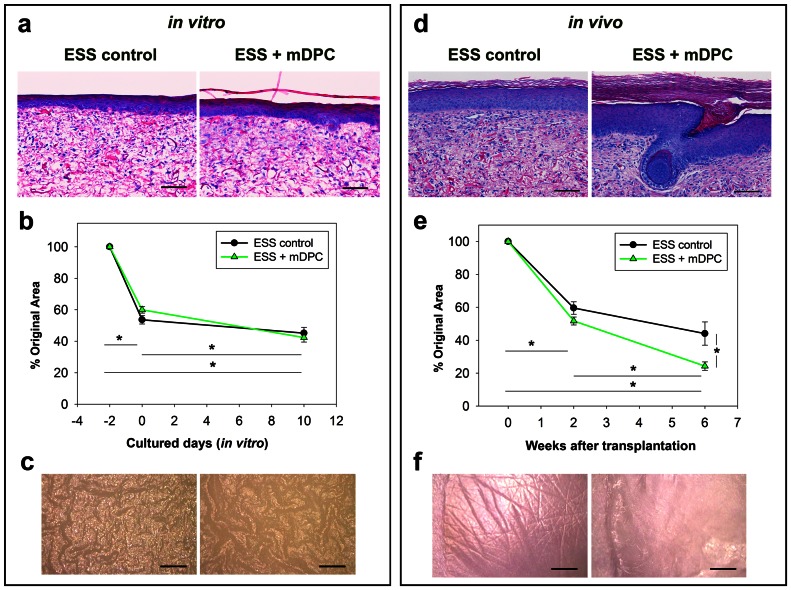
Evaluation of ESS *in vitro* and grafted ESS *in vivo* **.** ESS controls and ESS with mDPC-GFP were examined *in vitro* (a–c) and *in vivo* (d–f). There were no morphological differences between ESS models *in vitro* (a). After grafting, hair follicles were observed in ESS with mDPC-GFP, and not in controls (d). Significant decreases in ESS area over time were observed *in vitro* (b) and *in vivo* (e). Differences between groups were only observed at 6 weeks after grafting (e). Magnified *en face* images of the ESS surface show similar convoluted surfaces in both conditions *in vitro* (c), but only ESS controls developed dermatoglyphics after grafting (f). Scale bars in (a, d) = 100 µm. Scale bars in (c, f) = 1 mm.

Comparison of the current ESS model (ESS controls) to a newly developed ESS model (ESS with mDPC-GFP) indicated very similar characteristics, *in vitro*. A well-stratified epidermis, accompanied by significant decrease of surface electrical capacitance of both ESS was demonstrated (**Figure 1a** and data not shown). Statistically significant differences in graft contraction were observed over time, although there was no discernible difference between the two models, *in vitro* (**Figure 1b**). Magnified photomicrographs of ESS *in vitro* illustrated a wrinkly appearance of the epithelial surface regardless of the presence or absence of mDPC-GFP (**Figure 1c**). On the other hand, striking differences were noted 6 weeks after grafting. ESS controls had overall morphology resembling NHS but lacking cutaneous appendages, whereas ESS with mDPC-GFP possess unusually thick *stratum corneum* (SC) and contain bulbous pegs and/or hair follicles (**Figure 1d**). Statistically significant differences in graft contraction were also demonstrated *in vivo*, with a higher degree in ESS with mDPC-GFP (**Figure 1e**). Interestingly, pronounced skin dermatoglyphics were observed in ESS controls, but not in ESS with mDPC-GFP or in the murine host skin (**Figure 1f**).

### Developmental Process of Hair Regeneration in ESS

**Figure 2:**
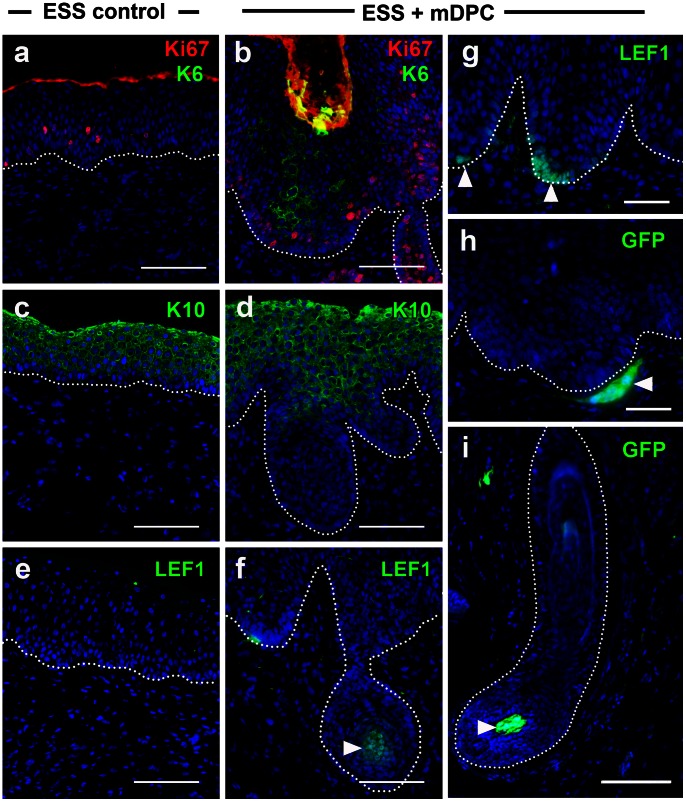
Characteristic features of hair induction *in vivo*. Compared to epithelium of ESS controls, which has fewer Ki67+ cells and no K6 staining (a), epithelium of ESS with mDPC-GFP contains numerous Ki67 with K6 expression (b). K10 stained positively in the suprabasal layer of ESS controls (c) and ESS with mDPC-GFP (d), but not the invaginating epidermis in ESS with mDPC-GFP (d). Similarly to embryonic development, LEF1 was restricted to the actively growing hair bulb (f, arrowhead) and placode (g, arrowheads). No nuclear LEF1 was observed in ESS controls (e). Corroborating these results, GFP+ dermal condensation was detected beneath the developing placode (h, arrowhead) and within the bulb (i, arrowhead). Dotted lines represent dermal-epidermal junctions. Scale bars = 100 µm.

Although both ESS models behave similarly *in vitro*, they were much different after grafting. One of the most prominent characteristics was the coincidence between epidermal cell proliferation and differentiation, which is generally observed in early hair development. Immunostaining of grafted ESS revealed high frequencies of Ki67+ cells in ESS with mDPC-GFP compared to fewer proliferating cells restricted to basal and spinous layers of ESS controls (**Figure 2b, 2a**). Keratin 6, previously found throughout the epidermis *in vitro*, was limited to the hair peg in ESS with mDPC-GFP, and was not detected in ESS controls (not shown and **Figure 2b, 2a**). Keratin 10, which was localized throughout the suprabasal layer of ESS controls (**Figure 2c**), was not found in the invaginating epidermis of ESS with mDPC-GFP (**Figure 2d**). In contrast to the murine host, in which synchronized follicular stages were initiated from the rostral to caudal regions, ESS with mDPC-GFP grafts, collected from the same region and the same time point, showed no synchronization of hair stages. LEF1+ cells were detected at the hair placode (**Figure 2g** **,** arrowheads), which was contacting a GFP+ dermal condensation (**Figure 2h** **,** arrowhead). Fully developed hair follicles containing GFP+ cells in the bulb were observed in the close proximity to these placodes (**Figure2i**). No LEF1 was detected in the interfollicular epidermis (**Figure 2e**).

### Sebaceous Gland and Skin Barrier Functions

**Figure 3:**
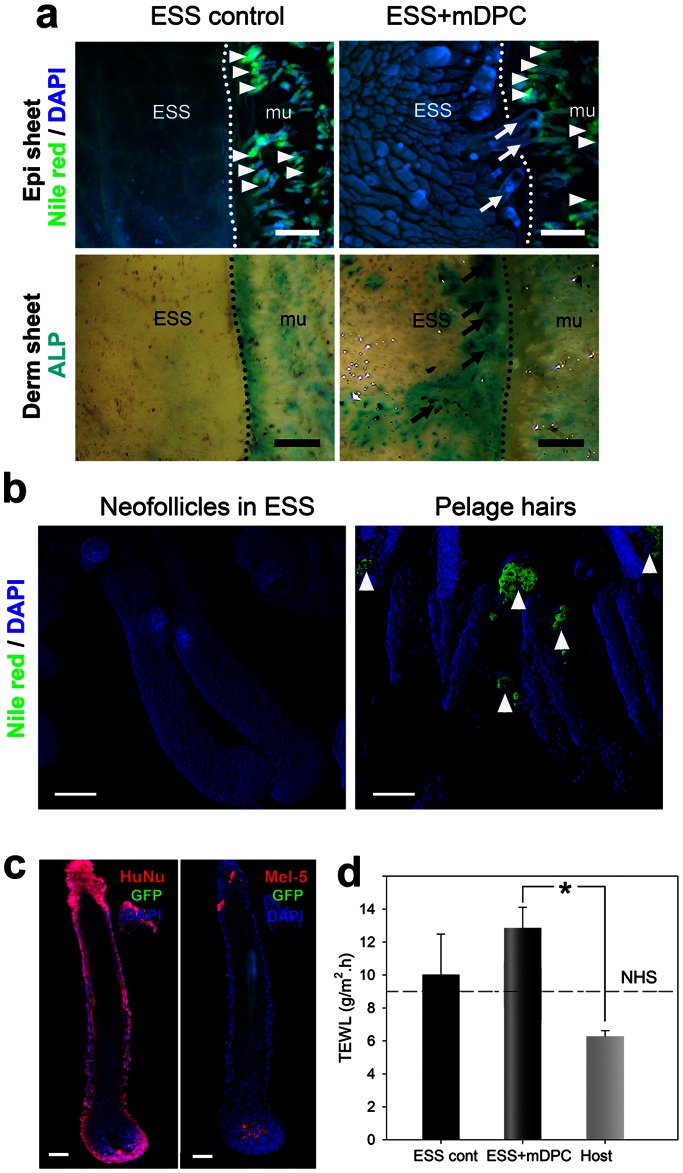
Absence of pilosebaceous units in chimeric ESS. Nile red and ALP stainings were performed on epidermis and dermis of ESS controls and ESS with mDPC-GFP (a). In ESS controls, epithelium was thin enough to visualize the skin surface lipids on the serosal side of the epidermis (a). Contrary to host skin containing sebaceous glands (arrowheads), no sebaceous glands were detected in ESS controls and ESS with mDPC-GFP. ALP in the dermis corresponded to the area where the hair follicles were situated (a, arrows). Close examination confirmed that neofollicles were deficient of sebaceous glands (b). On the other hand, sebaceous glands (white arrowheads) were observed above the bulge regions of pelage hairs. Immunohistochemistry confirmed the human origin (HuNu) and demonstrated no co-localization between Mel-5 and GFP in the bulb (c). Skin barrier integrity was evaluated by TEWL (d). Significantly higher TEWL was observed in ESS with mDPC-GFP compared to host skin, but not different from human volunteers (NHS). Scale bars in (a) = 500 µm; (b) = 100 µm and (c) = 50 µm.

While no hair shafts erupted through the skin surface, fully differentiated pigmented hair follicles were observed in the epidermal sheet of ESS with mDPC-GFP (**Figure 3a** **,** white arrows). ALP staining of the corresponding dermal surface indicated the area where the hair follicles were removed (**Figure 3a**, black arrows). In ESS controls, relatively smooth and thin epidermis showed dermatoglyphics filled with lipids that were positive for Nile red (**Figure 3a**). Contrary to host pelage hairs (**Figure 3b**, right) or to dissociated newborn murine-only ESS (data not shown), no sebaceous glands were associated with regenerated hairs in ESS (**Figure 3b** **,** left). The human origin, confirmed by human nuclei immunostaining, demonstrated positive labeling throughout the hair follicle except for the papillary region (**Figure 3c**). The origin of cells contributing to pigmented hair was investigated using Mel-5. The majority of Mel-5+ cells resided in the proximity of the hair bulb, whereas a few Mel-5+ cells were localized in the outer root sheath adjacent to the bulge (**Figure 3c**). No co-localization of Mel-5 and GFP was detected, suggesting that melanocytes were of human origin. To further elucidate skin barrier function of these ESS models, TEWL measurement of human ESS controls, chimeric ESS and host murine skin were compared to human skin (**Figure 3d**). While comparable TEWL was observed among ESS controls, NHS and host murine skin, significantly higher TEWL was observed in ESS with mDPC-GFP compared to skin of the murine host.

### Comparative Analyses of Gene and Morphological Differences among ESS Models

**Figure 4:**
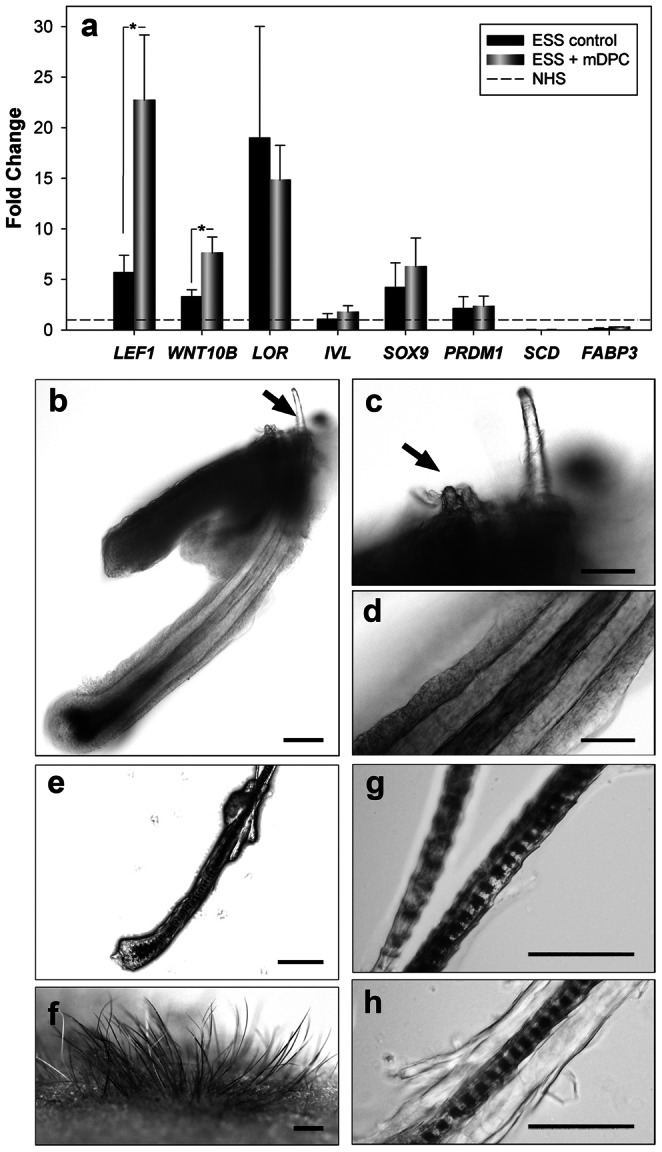
Comparison of ESS models. Gene expression analyses of selected genes involved in Wnt/β-catenin pathway, fatty acid metabolism and skin cornification, including *LEF1, WNT10B, LOR, INV, SOX9, PRDM1, SCD* and *FABP3*, were compared between ESS controls and ESS with mDPC-GFP after normalization to NHS (a). Asterisks represent statistically significant differences between the ESS groups (*p*<0.05). Morphological comparison of regenerated hair follicles in ESS with mDPC-GFP (b–d) and in newborn murine ESS was demonstrated (e–h). Scale bars = 50 µm, except in (b, e) = 100 µm and in (f) = 1 mm.

Expression of genes involved in trichogenesis, adipogenesis as well as genes that may be related to hair canal development were investigated (**Figure 4a**). Significant increases of *LEF1* and *WNT10B* expression were detected in ESS with mDPC-GFP compared to ESS controls. *SCD* and *FABP3* were expressed at very low levels in both ESS compared to NHS. *IVL, SOX9* and *PRDM1 (Blimp-1)* expression was found to be the same in all groups analyzed, whereas a remarkable increase in *LOR* expression was observed in both ESS models compared to NHS.

To investigate whether regenerated hairs contain keratinized hair shafts, the SC of grafted ESS was removed by tape stripping. No hair shafts were exposed to the skin surface after SC removal (not shown), suggesting that if the shafts existed, they may be located deeper in the epidermis. To prove this, the outer layer of the epidermis was gently removed by microdissection. Very fine vellus-like hairs, growing intra-epidermally, were exposed to the skin surface (**Figure 4b** **,** arrow). Small bulbous pegs adjacent to a large pigmented hair also possessed hair shafts (**Figure 4c** **,** arrow). Detailed examination indicated a typical arrangement of the outer root sheath (ORS) and inner root sheath, resembling native human hair (**Figure 4d**). In contrast, smaller regenerated pigmented hairs in grafted newborn murine ESS, erupted readily (**Figure 4e, f**), and possessed a pronounced striated medulla with a single cuticle layer (**Figure 4g, h**).

**Figure 5:**
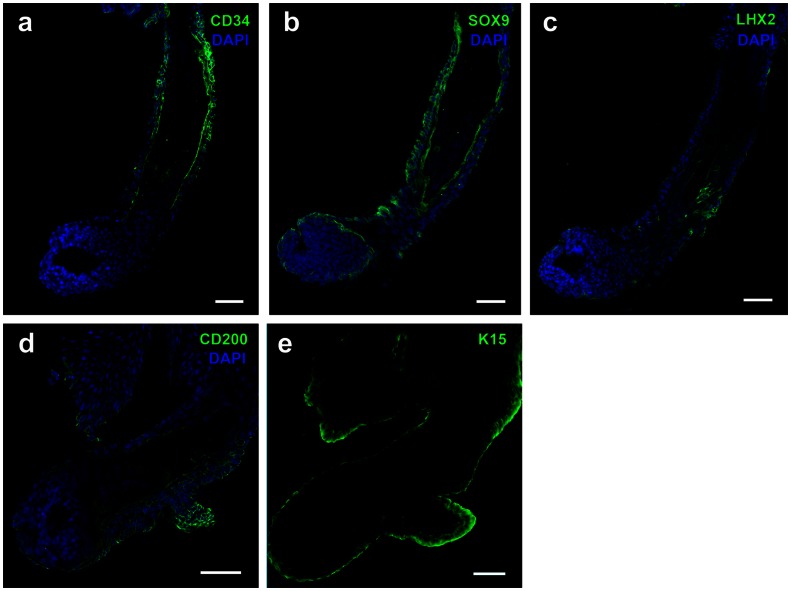
Immunostaining of regenerated hair follicles. Indirect immunohistochemistry was performed on frozen sections of microdissected hair follicles using antibodies against (a) CD34, (b) SOX9, (c) LHX2, (d) CD200 and (e) K15, respectively. Alexa Fluor 488 (green) was used to localize selected molecular markers before counterstaining of nuclei with DAPI (blue). Scale bars = 50 µm.

Immunostaining of regenerated hair follicles for CD34, SOX9, LHX2, CD200 and K15 that have been indicated as bulge stem cell markers was also performed to evaluate whether hair stem cells were present in this model. In this study, CD34, SOX9 and K15 were detected ubiquitously along the ORS of regenerated hair follicle, whereas LHX2 and CD200 were localized only to the proximal ORS (**Figure 5**).

## Discussion

Our long-term goal is to use tissue engineering to develop skin substitutes with greater homology to native skin, which will reduce morbidity in burn patients. As a step toward accomplishing this goal, we investigated how hair follicles form in ESS. This information will ultimately guide subsequent development of ESS with appendages. In this study, feasibility of hair restoration in tissue engineered skin by the use of expanded cultured cells was demonstrated. Regenerated hair follicles have morphology resembling native human hair, but lack sebaceous glands and do not readily erupt through the epidermal surface. Although not present during the *in vitro* incubation, pronounced dermatoglyphics filled with cutaneous lipids were found in ESS controls after grafting. In contrast, grafted ESS with regenerated hair follicles had a rough thick surface, similar to that of glabrous skin.

Interestingly, fully pigmented hairs were observed underneath the epidermal sheet of grafted ESS with mDPC-GFP. Presence of GFP-negative melanocytes in the bulb is believed to be the passenger human melanocytes which persisted in keratinocyte cultures as previously described [10]. This observation supports previous findings that suggest another role of dermal papilla and germinative matrix cells in guiding melanocyte migration [11], [12]. In addition to pigmented hairs, numerous small non-pigmented hair follicles and hair pegs were also observed. While punctate ALP staining patterns were only observed in grafted ESS with mDPC-GFP, high ALP activity was detected along the wound edges of both ESS with mDPC-GFP and ESS controls, in agreement with a previous report indicating the promotion of host animals’ hairs along the wound edge following skin transplantation [13]. Induction of anagen hairs in host animals as well as the more advanced stage of neofollicles adjacent to the wound edge suggests the plausible involvement of the wound-induced environment and hair growth. It is also possible that ingrowth of nerve fibers from the wound edge may promote maturation of nascent hair follicles.

In this study, both ESS models were deficient in sebaceous glands. Nonetheless, deposition of skin surface lipids along the dermatoglyphics of ESS controls may be attributed to the maintenance of barrier homeostasis. Cutaneous lipids may also be present in ESS with hair follicles, although they were not readily observed due to the unusually thick ridges of the skin. Hypoplastic sebaceous glands and thick scaling epidermis with increased cutaneous permeability have been previously reported in scarring alopecia and in asebia-*2J* mice [14]. To assess whether a similar phenotype existed in these grafted ESS lacking sebaceous glands, TEWL measurements were performed. Contrary to an exceptionally high TEWL in asebia-*2J* mice, there were no differences between grafted ESS and native human skin. The only significant difference detected was the TEWL between ESS with mDPC-GFP and host murine skin. Very low TEWL in skin of athymic mice has been reported previously [15]. These results suggested no compromise of skin integrity in either of the ESS models. It is also possible that sebum from adjacent murine skin accumulated on the dermatoglyphics of grafted human engineered skin, and contributed in maintenance of barrier integrity.

To better understand the mechanisms that govern trichogenesis, adipogenesis and hair canal formation, expression analyses of grafted ESS were compared to native human skin. While the upregulation of the Wnt/β-catenin pathway has been reported to be involved in hair morphogenesis and cycling [16]–[19], several signaling molecules in this pathway have also been implicated to inhibit adipogenesis [20], [21]. Aberrant signaling by TCF/lef-1 in K14ΔNlef1 transgenic mice induced sebaceous gland hyperplasia as well as ectopic sebaceous gland formation [22]–[24]. Furthermore, functional mutation of TCF/lef-1 has been demonstrated to alter lipid metabolism that ultimately resulted in skin barrier defects [25]. In this study, significant increase of *LEF1* and *WNT10B* expression was demonstrated in grafted ESS with mDPC-GFP compared to ESS controls, in which mRNA transcripts were several fold higher compared to native human skin. Unusually high *LEF1* and *WNT10B* gene expression may contribute to the deficiency of sebaceous glands in grafted ESS. Involvement of WNT10B in adipogenesis has been previously demonstrated in 3T3-L1 preadipocytes [26]. Exogenous recombinant WNT10B in 3T3-L1 cells stabilized β-catenin and blocked adipogenesis [26]. In agreement with these results, *SCD* and *FABP3* genes, which are expressed abundantly in sebaceous glands of native human skin, were either not detected or detected at very low levels in ESS. Interestingly, similar gene expression levels of *PRDM1* (*Blimp-1*) and *SOX9* were found in grafted ESS models (with or without nascent hairs) and in native human skin. This result suggests the possibility of sebaceous gland progenitor cells in grafted ESS. However, recent reports provided new evidence suggesting Blimp-1 as a terminal differentiation marker [27], [28], rather than sebaceous gland marker as previously shown [29], [30]. Had the sebaceous gland progenitor cells been present in ESS, it would be logical to deduce that upregulation of Wnt/β-catenin signaling, particularly in ESS with mDPC-GFP, strongly shifts the system to promote trichogenesis at the expense of sebaceous gland formation. Furthermore, proper balance of these signaling molecules may help to regenerate complete pilosebaceous units in ESS. Nonetheless, without concrete information supporting Blimp-1 and SOX9 as the sebaceous gland progenitor cell markers, definite conclusions cannot be drawn from these data. In addition to the possible role of sebaceous glands in emergence of the hair shaft, Sharov *et al* reported that transgenic mice with knockout of the gene for *matrix metalloproteinase 9* (*MMP-9*) exhibited delay of hair emergence through the epidermal surface [31]. Together with the evidence that sebaceous lipids are related to emergence of the hair shaft, the involvement of MMP-9, and possibly other degradative enzymes, in the mechanism of hair emergence demonstrates the complexity of the factors that participate in the regulation of normal hair development, phenotype, and function. Careful consideration will be given to the role of proteinases in hair shaft emergence in our future studies.

To investigate whether hair follicle stem cells existed in this ESS model, immunostaining was performed on the regenerated hair follicles using several molecular markers, including CD34, SOX9, LHX2, CD200 and K15. Although CD34, SOX9 and K15 were detected throughout ORS, punctate staining of CD200 and LHX2 was found only in proximal ORS suggesting a stem cell phenotype. CD34 is a hematopoietic stem cell marker that has been previously reported to be specific for murine hair stem cells. Nonetheless, a recent report suggested that CD34 is not as specific as CD200 for detection of human bulge stem cells [32]. K15 was found at normal levels of non-productive hair follicles of bald scalp from men with androgenic alopecia, but CD34 and CD200 were markedly diminished suggesting a failure of the conversion from K15-positive stem cells to CD34- and CD200-positive hair progenitor cells [33]. K15 and CD200 were also characterized as positive markers for keratinocyte stem cells of the hair follicle bulge area by Kloepper and co-workers, whereas the bulge was characterized as negative for CD34, connexin 43 and nestin [34]. These important distinctions identify some of the highly-specialized phenotypes of hair follicle keratinocytes that correspond with the normal anatomy and physiology of hair follicle development.

Although many reports have indicated the involvement of sebaceous glands in hair canal development, most, if not all, of the experiments were conducted in sheep and rodents [35], [36]. In agreement with these reports, hair shaft emergence was readily observed in grafted dissociated neonatal murine-only ESS. Considering that their pilosebaceous units were not developed until after birth, results should be interpreted cautiously. In human fetal development, complete pilosebaceous units formed around 20–21 weeks of gestational age [37]. Early establishment of human hair canals was observed intra-epidermally prior to initial eruption of vellus hairs [37]–[39]. Although results from this study suggested that incomplete hair canal formation in ESS may be due to the lack of sebaceous glands, we could not rule out the unusual cornification, which occurred after complete epidermal stratification. The sequence of hair and skin keratinization may also play a key role in human hair canal development. While future studies may need to emphasize developing ESS under submerged, instead of air-exposed, conditions to promote the proper order of hair and skin keratinization, this study provides a practical model for mechanistic studies of hair development in human epidermis of engineered skin.
